# Draft genome sequence of *Latilactobacillus sakei* 7-1 isolated from river water in China

**DOI:** 10.1128/mra.00384-26

**Published:** 2026-07-10

**Authors:** Shi Zhen, Taya Tang, Jørgen J. Leisner, Heng Li

**Affiliations:** 1 Changshu Hospital Affiliated to Soochow University, Changshu No.1 People's Hospital, Soochow University 576492, Changshu, China; 2 China-Mongolia Biomacromolecule Application “Belt and Road” Joint Laboratory, Inner Mongolia Agricultural University 117454, Hohhot, China; 3 Department of Veterinary and Animal Sciences, Faculty of Health and Medical Sciences, University of Copenhagen, Frederiksberg, Denmark; University of Maryland School of Medicine, Baltimore, Maryland, USA

**Keywords:** *Latilactobacillus sakei*, genome sequence, Illumina sequencing, freshwater

## Abstract

Here, we report the draft genome sequence of *Latilactobacillus sakei* 7-1, isolated from a river water sample in China, determined using Illumina sequencing. Phylogenomic analysis places the isolate within the *L. sakei* clade. This study demonstrates that this species is readily isolated as a free-living organism from freshwater environments.

## ANNOUNCEMENT

*Latilactobacillus sakei* is found in diverse environments, including various foods and the intestinal tracts of freshwater fish (1–4). However, information on this species as a free-living organism in freshwater ecosystems remains scarce (5).

The *L. sakei* isolate was obtained from the Baihe (白河) River near Wancun (湾村) village in northern Beijing, China, on 17 November 2024. An unfiltered, undiluted surface water sample was incubated in all-purpose Tween (APT) broth (Difco, Sparks, MD, USA) under anaerobic conditions at room temperature for 3 days. The culture was then transferred to nitrite polymyxin agar (NP agar), consisting of APT agar as the basal medium (Merck, Darmstadt, Germany) supplemented with 1 mL of 12% sodium nitrite (Ampliqon A/S, Odense, Denmark) and 0.2 mL of polymyxin supplement (Oxoid SR 099E) per 200 mL of agar, and incubated anaerobically at room temperature. Gram-positive, catalase-negative colonies were restreaked on APT agar for genomic analysis.

Genomic DNA from isolate 7-1 was extracted and purified using the HiPure Bacterial DNA Extraction Kit (D3146, Guangzhou Meiji Biotechnology). A sequencing library was constructed using the Vazyme TruePrep DNA Library Construction Kit (TD501, Nanjing Novizan). DNA quality was assessed using a NanoDrop ND-1000 spectrophotometer and 1.0% agarose gel electrophoresis. Whole-genome sequencing was performed on an Illumina HiSeq 4000 platform at Honsunbio, generating 8,824,835 paired-end read pairs totaling 2,647,450,500 bases. Raw paired-end reads were processed with fastp v1.3.0 (6) using default filtering and adapter-trimming settings, with no adapter sequences specified manually. *De novo* assembly was performed using SPAdes v4.2.0 (7) in isolate mode. Contigs shorter than 200 bp were excluded, and sequences flagged by the NCBI Foreign Contamination Screen were removed or trimmed before final genome submission. The initial assembly was assessed with QUAST v5.2.0 (8). Final assembly metrics and raw-read statistics were summarized using seqkit v2.13.0 (9). The final draft genome assembly comprised 188 contigs totaling 2,020,115 bp, with an *N*_50_ of 142,932 bp, a largest contig of 279,514 bp, and a GC content of 41.23%. The assembly was annotated using the NCBI Prokaryotic Genome Annotation Pipeline (PGAP) (10), which identified 2,090 genes, including 2,000 protein-coding genes, 54 tRNA genes, 11 rRNA genes, 3 ncRNA genes, and 22 pseudogenes. antiSMASH v8.0.4 (11) predicted three putative secondary-metabolite regions, but no well-characterized bacteriocin biosynthetic gene cluster was detected. For average nucleotide identity (ANI) analysis, the assembled genome was compared against 14 closely related reference genomes from GenBank using fastANI v1.34 (12); the resulting ANI matrix was clustered and visualized as a heatmap using Python with pandas (13), NumPy (14), and Matplotlib (15).

**Fig 1:**
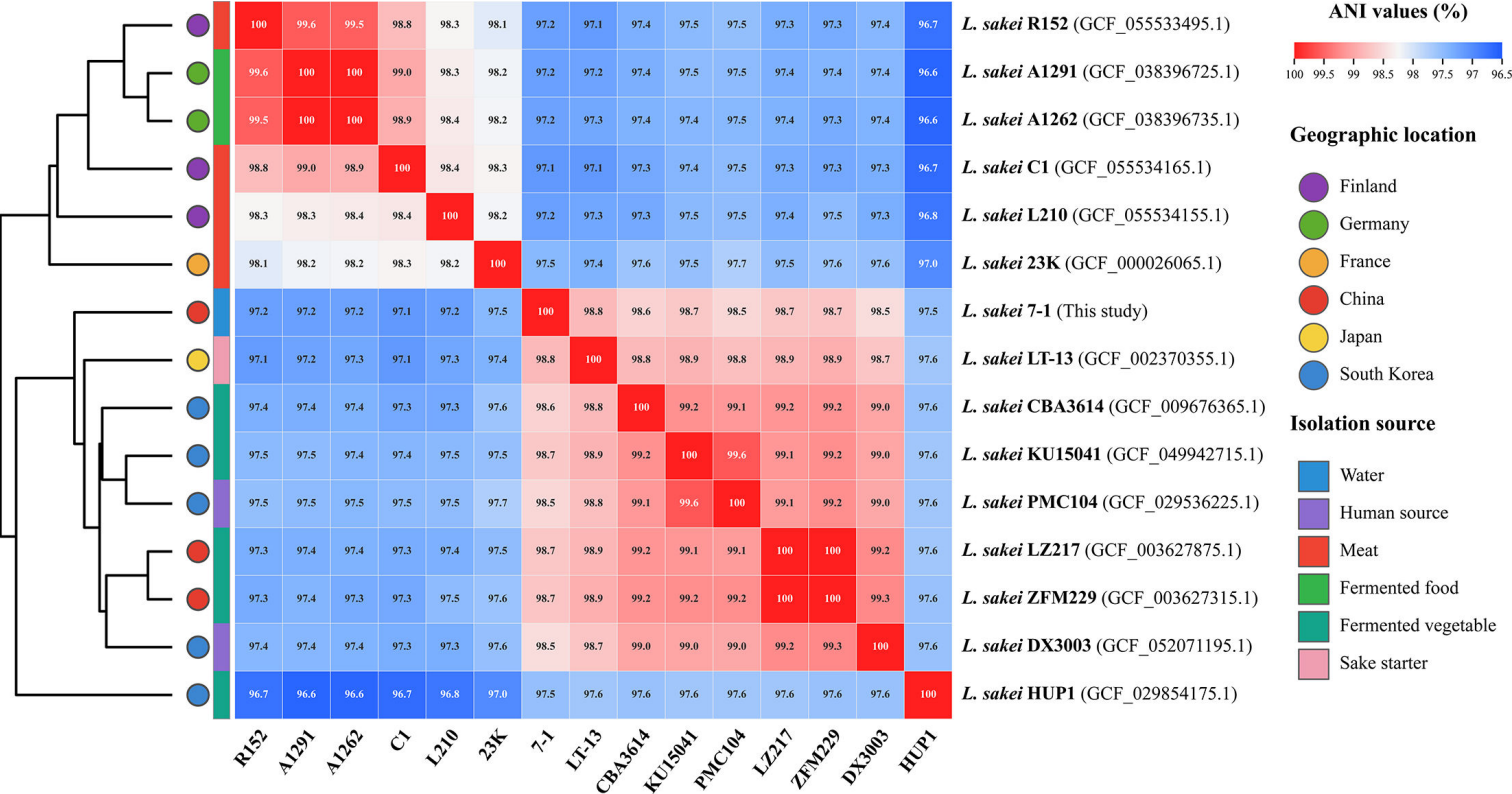
Average nucleotide identity (ANI) heatmap based on genome comparison of isolate 7-1 with 14 reference genomes.

The pairwise ANI heatmap illustrated that the freshwater isolate 7-1 clustered within the *L. sakei* clade (Fig. 1). Further comparison showed that 7-1 was most closely related to strain LT-13 (ANI, 98.835%), isolated from a Japanese sake starter. These findings support the classification of isolate 7-1 as *L. sakei* and expand the available genomic information for this species from freshwater-associated environments.

## Data Availability

Raw sequence reads have been deposited in the NCBI Sequence Read Archive under BioSample accession SAMN56648257 and SRA accession SRR37729657. The draft genome assembly has been deposited in GenBank under WGS accession JBYPRN000000000.
